# Posterior Reversible Encephalopathy Syndrome in a Postpartum Preeclamptic Woman without Seizure

**DOI:** 10.1155/2014/657903

**Published:** 2014-01-30

**Authors:** Ülkü Mete Ural, Gülsah Balik, Şenol Şentürk, Işık Üstüner, Uğur Çobanoğlu, Figen Kır Şahin

**Affiliations:** Department of Obstetrics & Gynecology, Recep Tayyip Erdoğan University School of Medicine, İslampaşa Mahallesi, 53100 Rize, Turkey

## Abstract

Posterior reversible encephalopathy syndrome (PRES) is a cliniconeuroradiological entity presenting with headache, confusion, visual disturbances or blindness, and seizures. Parieto-occipital white matter changes due to vasogenic oedema can be observed on imaging modalities. It rarely occurs without seizures and after delivery. We report a 33-year-old multigravida with a history of preeclampsia in term pregnancy complicated by PRES without seizures at the postpartum period. Clinical improvement with complete resolution without any complications was observed on the 6th day after delivery. Posterior reversible encephalopathy syndrome is reversible when early diagnosis is established and appropriate treatment is started without delay.

## 1. Introduction

Posterior reversible leukoencephalopathy syndrome (PRES) is a clinical and radiological syndrome which is also known as reversible posterior cerebral edema syndrome, hyperperfusion encephalopathy, or brain capillary leak syndrome [1].

PRES has been accompanied by a number of medical conditions such as hypertensive encephalopathy, preeclampsia, eclampsia, acute or chronic renal diseases, hemolytic uremic syndrome, use of cytotoxic and immunosuppressant drugs, blood transfusion, and electrolyte disturbances [2]. Preeclampsia and eclampsia are among the most common causes of PRES. Prompt recognition and treatment are crucial to avoid the permanent damage leading to sequelae and even mortality.

Clinical and radiological features of PRES include headache, encephalopathy, seizures, cortical visual changes, and parieto-occipital white matter edema visualized on neuroimaging modalities [3]. Even though PRES is increasingly recognized and reported in the current literature, its incidence is still obscure. In this case report, we present a preeclamptic pregnant woman complicated by PRES after delivery without seizures.

## 2. Case Report

**Figure 1:**
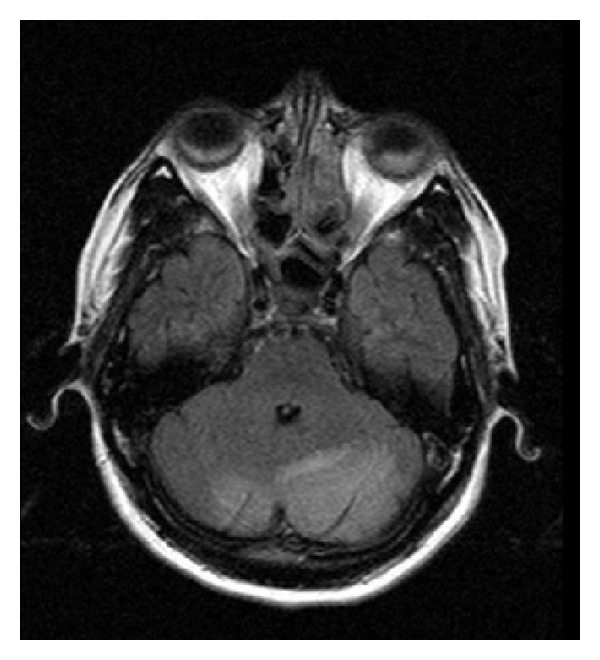
MRI: hyperintense signal involving the white matter typically in occipital regions.

**Figure 2:**
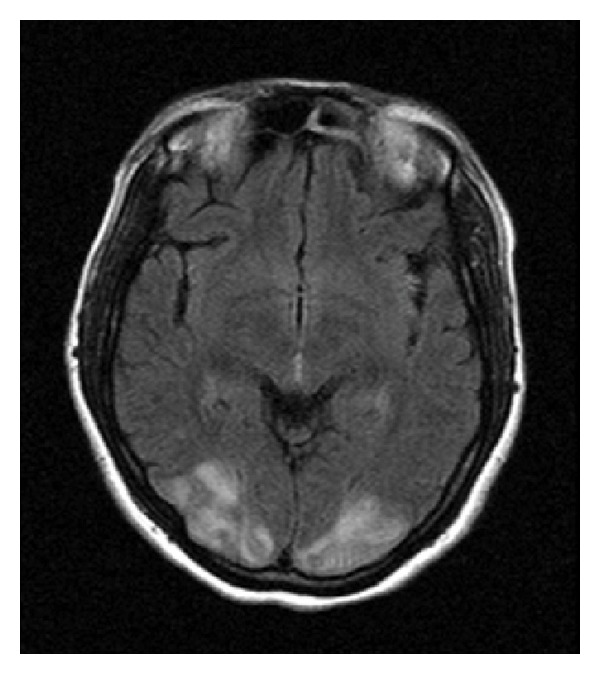
FLAIR axial images: hyperintense signal involving the white matter typically in occipital regions.

**Figure 3:**
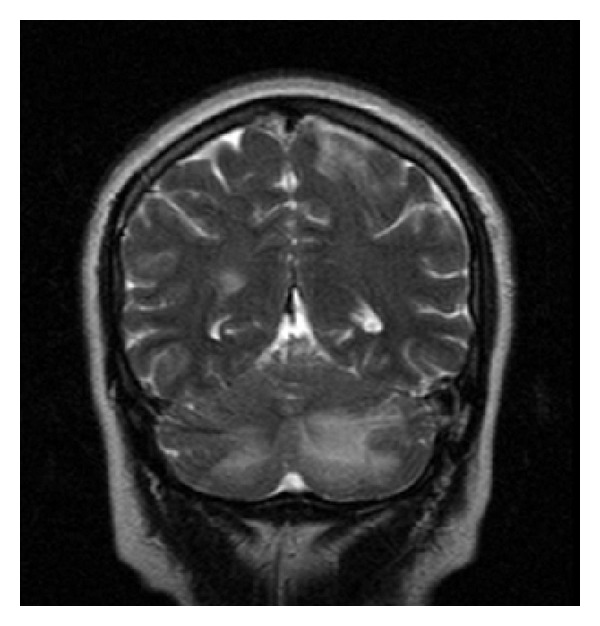
MRI, T_2_-weighted coronal image: hyperintense signal on bilateral occipital and cerebellar regions.

A 33-year-old multigravida at the 38th week of gestation was admitted to our clinic with complaints of headache. She had no visual abnormalities and her blood pressure was 210/120 mmHg with pulse rate of 108/minute. Urinalysis revealed 2+ proteinuria and she had bilateral pedal oedema. There was no past history of hypertension, vision abnormalities, seizures, or any other pathologies. Complete blood count revealed a hemoglobin level of 9.1 g/dL and a platelet count of 162.000/mm^3^. Renal and hepatic function tests as well as electrocardiogram were within normal limits. Magnesium sulphate 4 g loading dose was administered intravenously, followed by continuous intravenous infusion at 2 g per hour. After labour induction, a 3100 g baby was born with normal vaginal delivery. She complained of blindness 13 hours after delivery. MRI brain images obtained after neurology consultation revealed hyperintense and FLAIR signal lesions extending beyond the occipital lobes and involved both cerebellar hemispheres (Figures 1, 2, and 3). Clinical and radiological findings were assumed to be consistent with PRES. Three days after delivery, her blood pressure was controlled and her visual symptoms nearly disappeared. Meanwhile, the patient had no episodes of seizures and on the 6th postpartum day, there was a complete recovery from blindness and she was discharged without any recurrent symptoms afterwards.

## 3. Discussion

Posterior reversible leukoencephalopathy syndrome is a rare and serious entity of central nerve system, characterized by headache, altered mental status, seizures, and visual loss. Seizures, which are usually generalized and tonic clonic, are often the presenting manifestation [4]. Our case has a unique presentation since it occurred without seizures in a preeclamptic woman. She only had visual loss occurring after delivery.

A pregnant woman presenting with hypertension and blindness at term constitutes a diagnostic dilemma. The possibilities that must be kept in mind include cerebrovascular hemorragia, eclampsia, and clinical syndromes like PRES. Hypertensive retinopathy, exudative retinal detachment, and cortical blindness are three common visual complications of preeclampsia and eclampsia. Currently, blindness in severe preeclampsia is more likely to be associated with cortical aetiology [5]. Visual abnormalities that accompany PRES include hemianopsia, visual neglect, auras, visual hallucinations, and cortical blindness. The visual loss is usually regained within a period of 4 hours to 8 days after treatment [6].

The pathophysiological mechanism underlying PRES is still vague. It may be related to disordered cerebral autoregulation and endothelial dysfunction. The combination of acute hypertension and endothelial damage can lead to vasogenic edema elicited by the forced leakage of serum through capillary walls and into the brain interstitium [7]. The reason for the primary involvement of posterior brain regions is not well understood. One possibility may be the regional heterogeneity of the sympathetic innervation of the intracranial arterioles. This is explained by better autoregulation of the anterior circulation due to better sympathetic innervations as compared to the posterior circulation [8]. Acute hypertension can lead to hyperperfusion and edema in the posterior circulation in PRES.

Neuroimaging is essential for the diagnosis of PRES and the radiological abnormalities encountered in PRES are best demonstrated by magnetic resonance imaging (MRI). MRI shows symmetrical white matter edema in the posterior cerebellar hemispheres that particularly involve the parieto-occipital regions bilaterally [9]. T_2_-weighted MRI shows areas of hyperintense signal and is thought to capture the images with the best quality, but fluid attenuated inversion recovery (FLAIR) sequences may improve detection of cortical-subcortical areas of injury and help distinguish vasogenic edema from cytotoxic edema. More severe radiological findings are more likely to be observed with more severe clinical pictures [10].

Owing to the heterogeneity of clinical settings and relative lack of data on PRES, treatment recommendations are somewhat limited. The initial goal in malignant hypertension is to rapidly lower the diastolic pressure to about 100 to 105 mmHg. More aggressive blood pressure control may reduce the blood pressure below the autoregulatory range and ischemic events such as stroke or coronary disease may exist [11]. In most cases of PRES, neurological symptoms and cerebral lesions disappear completely within days to weeks after control of blood pressure.

Recommendations for the treatment of eclampsia differ from PRES in other clinical settings. Delivery of the baby and placenta is often sufficient. Magnesium therapy should be initiated as soon as eclampsia or PRES in pregnancy is suspected, as it treats both seizures and hypertension [12].

The prognosis of PRES is usually benign. Regardless of etiology, hypertension is a feature in the vast majority of PRES patients. Most investigators believe that hypertensive encephalopathy and preeclampsia share similar mechanisms. Clinical improvement always follows the treatment of elevated blood pressure.

Posterior reversible leukoencephalopathy syndrome may be a diagnostic and therapeutic challenge when it develops in eclampsia and preeclampsia. Preeclampsia and eclampsia may be the most common causes of PRES. This syndrome is generally characterized by seizures but visual loss occurring in a preeclamptic woman must remind PRES even if seizures do not accompany the clinical scene. In our case, no focal deficits or visual abnormalities were detected before the delivery. We especially want to emphasize that PRES can occur at the postpartum period without seizure. In most cases of PRES, neurological symptoms and cerebral lesions disappear with treatment. Control of blood pressure is vital to avoid irreversible damage to central nervous system.

## References

[1] Calabrese LH, Dodick DW, Schwedt TJ, Singhal AB (2007). Narrative review: reversible cerebral vasoconstriction syndromes. *Annals of Internal Medicine*.

[2] Stott VL, Hurrell MA, Anderson TJ (2005). Reversible posterior leukoencephalopathy syndrome: a misnomer reviewed. *Internal Medicine Journal*.

[3] Bartynski WS, Boardman JF (2007). Distinct imaging patterns and lesion distribution in posterior reversible encephalopathy syndrome. *American Journal of Neuroradiology*.

[4] Kastrup O, Gerwig M, Frings M, Diener H-C (2012). Posterior reversible encephalopathy syndrome (PRES): electroencephalographic findings and seizure patterns. *Journal of Neurology*.

[5] Lee VH, Wijdicks EFM, Manno EM, Rabinstein AA (2008). Clinical spectrum of reversible posterior leukoencephalopathy syndrome. *Archives of Neurology*.

[6] Thackeray EM, Tielborg MC (2007). Posterior reversible encephalopathy syndrome in a patient with severe preeclampsia. *Anesthesia and Analgesia*.

[7] Schwartz RB, Feske SK, Polak JF (2000). Preeclampsia-eclampsia: clinical and neuroradiographic correlates and insights into the pathogenesis of hypertensive encephalopathy. *Radiology*.

[8] Bartynski WS (2008). Posterior reversible encephalopathy syndrome—part 1: fundamental imaging and clinical features. *American Journal of Neuroradiology*.

[9] Lamy C, Oppenheim C, Méder JF, Mas JL (2004). Neuroimaging in posterior reversible encephalopathy syndrome. *Journal of Neuroimaging*.

[10] Fugate JE, Claassen DO, Cloft HJ, Kallmes DF, Kozak OS, Rabinstein AA (2010). Posterior reversible encephalopathy syndrome: associated clinical and radiologic findings. *Mayo Clinic Proceedings*.

[11] Roth C, Ferbert A (2010). Posterior reversible encephalopathy syndrome: long-term follow-up. *Journal of Neurology, Neurosurgery and Psychiatry*.

[12] Striano P, Striano S, Tortora F (2005). Clinical spectrum and critical care management of posterior reversible encephalopathy syndrome (PRES). *Medical Science Monitor*.

